# Prevalence and Characteristics of Non–US-Born and US-Born Health Care Professionals, 2010-2018

**DOI:** 10.1001/jamanetworkopen.2021.8396

**Published:** 2021-04-29

**Authors:** Yvonne Commodore-Mensah, Kelli DePriest, Laura J. Samuel, Ginger Hanson, Rita D’Aoust, Eric P. Slade

**Affiliations:** 1 Johns Hopkins School of Nursing, Baltimore, Maryland; 2 Department of Epidemiology, Johns Hopkins Bloomberg School of Public Health, Baltimore, Maryland

## Abstract

**Importance:**

Immigration to the US results in greater racial/ethnic diversity. However, the contribution of immigration to the diversity of the US health care professional (HCP) work force and its contribution to health care are poorly documented.

**Objective:**

To examine the sociodemographic characteristics and workforce outcomes of non–US-born and US-born HCPs.

**Design, Setting, and Participants:**

This cross-sectional study used national US Census Bureau data on US-born and non–US-born HCPs from the American Community Survey between 2010 and 2018. Demographic characteristics and occupational data for physicians, advanced practice registered nurses, physician assistants, registered nurses, licensed practical nurses or licensed vocational nurses, and other HCPs were included for analysis. Data were analyzed between December 2020 and February 2021.

**Exposures:**

Nativity status, defined as US-born HCP vs non–US-born HCP (further stratified by <10 years or ≥10 years of stay in the US).

**Main Outcomes and Measures:**

Annual hours worked, proportion of work done at night, residence in medically underserved areas and populations, and work in skilled nursing/home health settings. Inverse probability weighting of 3 nativity status groups was carried out using logistic regression. *F* test statistics were used to test across-group differences. Data were weighted using American Community Survey sampling weights.

**Results:**

Of a total of 657 455 HCPs analyzed (497 180 [75.5%] women; mean [SD] age, 43.7 [13.0] years; 518 317 [75.6%] White, 54 233 [10.8%] Black, and 60 680 [9.6%] Asian), non–US-born HCPs (105 331 in total) represented 17.3% (95% CI, 17.2%-17.4%) of HCPs between 2010 and 2018. They were older (mean [SD] age, 44.7 [11.6] years) and had more education (75 227 [70.1%] HCPs completed college) compared with US-born HCPs (mean [SD] age, 43.4 [13.3] years; 304 601 [55.2%] completed college). Nearly half of non–US-born HCPs (47 735 [43.0%]) were Asian. In major metropolitan areas, non–US-born HCPs represented 40% or more of all HCPs. Compared with US-born HCPs, non–US-born HCPs with less than 10 years and 10 or more years of stay worked 32.3 hours (95% CI, 19.2 to 45.4 hours) and 71.6 hours (95% CI, 65.1 to 78.2 hours) more per year, respectively. Compared with US-born HCPs, non–US-born HCPs were more likely to reside in areas with shortages of health care professionals (estimated percentage: <10 years, 75.3%; ≥10 years, 62.8% vs US-born, 8.3%) and work in home health settings (estimated percentage: <10 years, 17.5%; ≥10 years, 13.1% vs US-born, 12.8%).

**Conclusions and Relevance:**

The contributions of non–US-born HCPs to US health care are substantial and vary by profession. Greater efforts should be made to streamline their immigration process and to harmonize training and licensure requirements.

## Key Points

**Question:**

How large is the non–US-born health care professional (HCP) work force in the US, what occupations are non–US-born HCPs more likely to hold, and how do their work conditions compare with US-born HCPs?

**Findings:**

In this cross-sectional study, 17.3% of HCPs were born outside the US. Overall, non–US-born HCPs worked more hours, were more likely to work at night and in skilled nursing and/or home health settings, and were more likely to reside in medically underserved areas than US-born HCPs. These associations differed by health care occupation and the length of stay in the US.

**Meaning:**

These findings suggest that non–US-born HCPs are making significant contributions to health care in the US.

## Teaser

This cross-sectional study uses data from the American Community Survey (ACS) to examine the prevalence of non–US-born health care professionals in the US health system between 2010 and 2018 and compares their work conditions with US-born health care professionals.

## Introduction

In 2018, more than 45 million immigrants lived in the US, accounting for approximately 14% of the population.^1^ Immigrants were most commonly from Mexico (25%), followed by India (6%) and China (5%).^1^ However, immigrants are increasingly coming from South Asia, Central America, and Africa in the past decade.^2^

Health disparities among racial/ethnic minority groups in the US are well established.^3,4^ Patient-level factors (such as mistrust and treatment refusal), health system–level factors (eg, language barriers, access to health care), and structural social determinants related to segregation and poverty contribute to these disparities.^4,5^ Patients who receive care from race-concordant health professionals rate their care as more participatory than patients who receive care from race-discordant health professionals.^6^ Expanding and diversifying the health workforce may improve access to care and reduce health disparities. The ongoing COVID-19 pandemic has further amplified longstanding health disparities.^7^ Throughout the pandemic, even as the disease spread from urban to rural areas,^8^ racial and ethnic minority populations experienced a disproportionate disease burden.^9^

As the US diversifies, the health workforce must adapt to meet the population’s needs. Immigrants help address health workforce shortages while furthering needed racial/ethnic diversity.^10^ A more diverse workforce is associated with higher patient satisfaction owing to shared language, cultural sensitivity, and a better appreciation of patients’ socioeconomic realities.^10,11^ Diversity among health care professionals (HCPs) has not kept pace with demographic changes in the US population. In 2018, only 5% of physicians^12^ and only 6.2% of nurses^13^ were Black, compared with 13.4% of the US population. The shortage of Hispanic physicians has worsened over the past 30 years^14^; only 6% of physicians in 2018^12^ and only 5% of nurses in 2017 were Hispanic.^13^

It is unclear to what extent immigrants constitute the current share of HCPs, and how they differ from US-born HCPs in employment outcomes. Thus, we examined the unique contributions of non–US-born HCPs by comparing their sociodemographic characteristics and employment outcomes with US-born HCPs to inform ongoing efforts to diversify the health workforce.

## Methods

### Design

The 2010 to 2018 American Community Survey (ACS) is an ongoing, nationally representative annual cross-sectional study of US-born and non–US-born adults residing in the 50 US states and the District of Columbia (excluding US territories).^15^ The ACS, described in detail elsewhere,^15^ obtains information on socioeconomic, housing, and demographic characteristics recorded on an online or paper survey from an invited sample of 1 in 38 US households. We extracted data for HCPs who participated in the survey. The initial sample included 718 507 HCPs whose primary job was in a health care delivery industry based on North American Standard Industry Classification System codes,^16^ including hospitals, nursing and residential care facilities, outpatient care, and home and other health care services. Professional roles were identified based on self-reported occupation using 2010 US Census occupation codes.^17^ Six groups of health care professionals were examined: registered nurses (RNs) with at least an associate degree; advanced practice registered nurses (APRNs; including nurse practitioners, certified nurse midwives, and certified registered nurse anesthetists with at least a master’s degree); licensed practical nurses (LPNs) and licensed vocational nurses (LVNs); physician assistants (PAs); physicians; and other health care professionals (eg, therapists, dietitians, paramedics, acupuncturists, medical technicians, pharmacists, sonographers, optometrists). A total of 61 052 observations for individuals who had not worked in the past 12 months were excluded, leaving a sample of 657 455 HCPs. The Johns Hopkins School of Nursing institutional review board deemed this study exempt from approval because of the use of deidentified public use data files. Participants in the ACS provided informed consent prior to participating in the survey.^16^ This study followed the Strengthening the Reporting of Observational Studies in Epidemiology (STROBE) reporting guideline.

### Measures

#### Dependent Variables

Four dependent variables were used as indicators of potential contributions to US health care by non–US-born HCPs: annual hours worked, working at night, residence in medically underserved areas and populations (MUA/P), and employment in a skilled nursing, residential, institutional, or home health setting (henceforth, *skilled nursing/home health setting*). Annual hours worked were calculated by multiplying weeks worked by reported usual hours worked per week in the past 12 months. An individual was classified as working at night if they reported arriving at work between 6:00 pm and 1:59 am. We identified MUA/P, geographic areas and populations with a lack of access to primary care services,^18^ through 2020 county-level records obtained from the Health Resources and Services Administration,^19^ which we then matched to the ACS data set based on county of residence using a crosswalk file from Integrated Public Use Microdata Series (IPUMS) USA.^20^

#### Independent Variable

The chief exposure of interest was nativity status, classified as US-born (referent), non–US-born with under 10 years of stay in the US, and non–US-born with 10 years or more of stay in the US. Among non–US-born, the year of US entry was used to calculate the length of stay, which was dichotomized at 10 years because it is a commonly used indicator of acculturation among immigrants.^21^ Countries of birth were grouped using a modified version of standardized world regions^22^: Mexico/Central America/Caribbean (ie, Mexico and all countries in Central America and the Caribbean Islands), South America, Europe, Russia, Africa, the Middle East, Asia, Southeast Asia, the Indian subcontinent, Australia/New Zealand, Canada, and US territories. The details of countries included in these regions are published elsewhere.^22^

### Statistical Analysis

Descriptive statistics for the full sample were weighted using ACS sampling weights. Nativity status was the main independent variable. An *F* test was used to test for trends in the percentage of non–US-born HCPs, comparing participants in the 2013-2015 and 2016-2018 surveys with participants in the 2010-2012 survey. Pooling the years reduced the within-period variance. Inverse probability weighting^23^ was used to balance the weighted sample distributions of the nativity status groups in relation to demographic characteristics, educational attainment, health care occupations, geographic location (ie, residence in metropolitan vs nonmetropolitan areas, US census region), and survey years. Inverse probability weighting of the 3 immigration groups was carried out using logistic regression. The weighted means of the dependent variables within each immigration group and their 95% confidence intervals were then estimated, and *F* test statistics tested across-group differences. Significance levels were adjusted for multiple comparisons using the Benjamini-Hochberg procedure.^24^ The false discovery rate was set at *P* < .05 using a 2-sided test. All the dependent variable means were estimated overall and stratified by health care occupation because we anticipated qualitative differences in outcomes based on profession. All statistical analyses were performed with Stata version 16 (StataCorp).

## Results

### Sociodemographic Characteristics of US-Born and Non–US-Born Health Care Professionals

From 2010 to 2018, non–US-born HCPs (105 331 in total) constituted an estimated 17.3% of all US HCPs (95% CI, 17.2%-17.4%). In 2018 alone, an estimated 1 411 000 non–US-born HCPs and 6 548 000 US-born HCPs worked in US health care settings. Each non–US-born HCP worked a mean of 1924 hours (mean [SD] annual work hours: <10 y stay, 1902.9 [754.4] hours; ≥10 y stay, 1942.2 [681.0] hours), implying that together, non–US-born HCPs contributed more than 2.7 billion hours worth of health care services in 2018 alone. Table 1 provides descriptive information about their sociodemographic characteristics, residential location, and region of origin. Among non–US-born HCPs, Asian participants, the largest racial/ethnic group, represented 43.0% (47 735 HCPs), and Southeast Asia was the region of origin for 24.6% (27 391 HCPs). Although Southeast Asia includes Hong Kong, Indonesia, Vietnam, Philippines, Singapore, Vietnam, Thailand, and other countries, most HCPs from Southeast Asia in 2018 were RNs (1582 [51.6%] HCPs) and 86.8% of Southeast Asian RNs (1328 HCPs) were born in the Philippines. Most non–US-born HCPs (77 043 [73.0%]) spoke a second language other than English at home compared with 32 652 (6.6%) of US-born HCPs. US-born HCPs (425 378 [78.0%]) were more likely to be women than non–US-born HCPs (71 802 [68.3%]). Notably, the larger population of RNs and LPNs/LVNs, who were mostly women, contributed to the gendered distribution of the health care workforce. Among the 20 metropolitan areas with the highest proportions of non–US-born HCPs, at least 40% of HCPs were non–US-born in 5 major metropolitan areas in California and in the New York-Newark-Jersey City metropolitan area in New York and New Jersey (Figure).

**Table 1.** Sociodemographic Characteristics of HCPs by Nativity Status, 2010-2018 American Community Survey^a^

Characteristic	HCPs, No. (%)					
	Overall	Non–US-born			US-born
		All	<10 y stay	≥10 y stay	
Total	657 455 (100)	105 331 (17.3)	15 338 (2.7)	89 993 (14.6)	552 124 (82.7)
Age, mean (SD), y	43.7 (13.0)	44.7 (11.6)	36.0 (8.3)	46.3 (11.6)	43.4 (13.3)
Female gender	497 180 (75.5)	71 802 (68.3)	10 387 (67.7)	61 415 (68.4)	425 378 (77.0)
Male gender	160 275 (24.4)	33 529 (31.8)		51 764 (57.5)	126 746 (23.0)
Education					
Associate’s degree	155 000 (22.6)	15 429 (14.7)	1457 (9.8)	13 972 (15.6)	139 571 (24.3)
Bachelor’s degree	183 362 (28.9)	34 241 (33.1)	6010 (38.4)	28 231 (32.1)	149 121 (28.0)
≥Master’s degree	196 466 (28.9)	40 986 (37.0)	5750 (36.6)	35 326 (45.9)	155 480 (27.2)
Race					
White	518 317 (75.6)	36 190 (33.2)	4118 (26.5)	32 072 (34.5)	482 127 (84.5)
Black/African American	54 233 (10.8)	14 419 (16.7)	2132 (17.0)	12 287 (16.6)	39 814 (9.5)
Asian	60 680 (9.6)	47 735 (43.0)	8187 (50.6)	39 538 (41.6)	12 945 (2.6)
Other^b^	24 225 (4.1)	6987 (7.1)	891 (5.9)	6096 (7.3)	17 238 (3.4)
Hispanic ethnicity	42 837 (7.5)	15 385 (15.5)	1956 (13.1)	13 429 (15.9)	27 452 (5.9)
Health care occupation					
Physicians	78 557 (11.4)	22 265 (19.8)	3302 (20.5)	18 963 (19.6)	56 292 (9.7)
APRNs	13 509 (1.9)	1282 (1.1)	66 (0.4)	1216 (1.3)	12 227 (2.1)
PAs	8797 (1.4)	1128 (1.1)	150 (1.0)	978 (1.1)	7669 (1.4)
RNs	239 293 (36.2)	37 207 (35.7)	5714 (36.6)	31 493 (35.5)	202 086 (36.3)
LPNs/LVNs	64 428 (10.0)	8458 (8.7)	1259 (9.0)	7199 (8.7)	55 970 (10.3)
Other HCPs	251 857 (38.9)	34 853 (33.4)	4823 (32.4)	30 030 (33.6)	217 004 (40.0)
Metropolitan area residence	497 237 (80.0)	98 629 (94.5)	14 370 (94.4)	84 259 (94.5)	398 608 (77.0)
Census region					
Northeast	132 496 (19.8)	26 235 (25.0)	3725 (24.3)	22 510 (25.1)	106 261 (18.8)
Midwest	152 760 (23.3)	12 322 (13.2)	2134 (15.8)	10 188 (12.7)	140 438 (25.5)
South	239 115 (36.4)	34 702 (33.1)	5448 (36.0)	29 254 (32.5)	204 413 (37.2)
West	133 084 (20.3)	32 072 (28.7)	4031 (23.9)	28 041 (29.6)	101 012 (18.6)
Foreign language spoken at home	109 695 (18.1)	77 043 (73.0)	12 942 (82.8)	64 101 (71.2)	32 652 (6.6)
Region of birth^c^					
Mexico/Central America/Caribbean	17 543 (3.2)	17 543 (18.3)	1719 (12.3)	15 824 (19.4)	NA
Southeast Asia	27 391 (4.3)	27 391 (24.6)	4351 (26.4)	23 040 (24.2)	NA
Indian subcontinent	12 760 (2.1)	12 760 (12.0)	2627 (17.0)	10 233 (11.0)	NA
Asia other	8851 (1.4)	8851 (7.9)	1312 (8.0)	7539 (7.9)	NA
Europe	14 816 (2.3)	14 816 (13.0)	1320 (8.1)	13 496 (14.0)	NA
Africa	8390 (1.7)	8390 (9.7)	1638 (12.9)	6752 (9.1)	NA
South America	4535 (0.8)	4535 (4.4)	659 (4.3)	3876 (4.4)	NA
Middle East	4235 (0.7)	4235 (4.0)	662 (4.4)	3573 (3.9)	NA
Canada	4022 (0.6)	4022 (3.4)	558 (3.4)	3464 (3.4)	NA
Australia/New Zealand	340 (0.05)	340 (0.3)	57 (0.3)	283 (0.3)	NA
US territories	2788 (0.5)	2788 (2.8)	492 (3.2)	2296 (2.7)	NA

Abbreviations: APRNs, advanced practice registered nurse; HCPs, health care professionals; LPNs, licensed practical nurses; LVNs, licensed vocational nurses; NA, not applicable; PAs, physician assistants; RNs, registered nurses.

^a^ Weighted using American Community Survey sampling weights.

^b^ Included American Indian/Alaskan Native, Native Hawaiian, Pacific Islander, and multiracial persons.

^c^ Countries of birth were grouped using a modified version of standardized world regions.^22^

**Figure.**
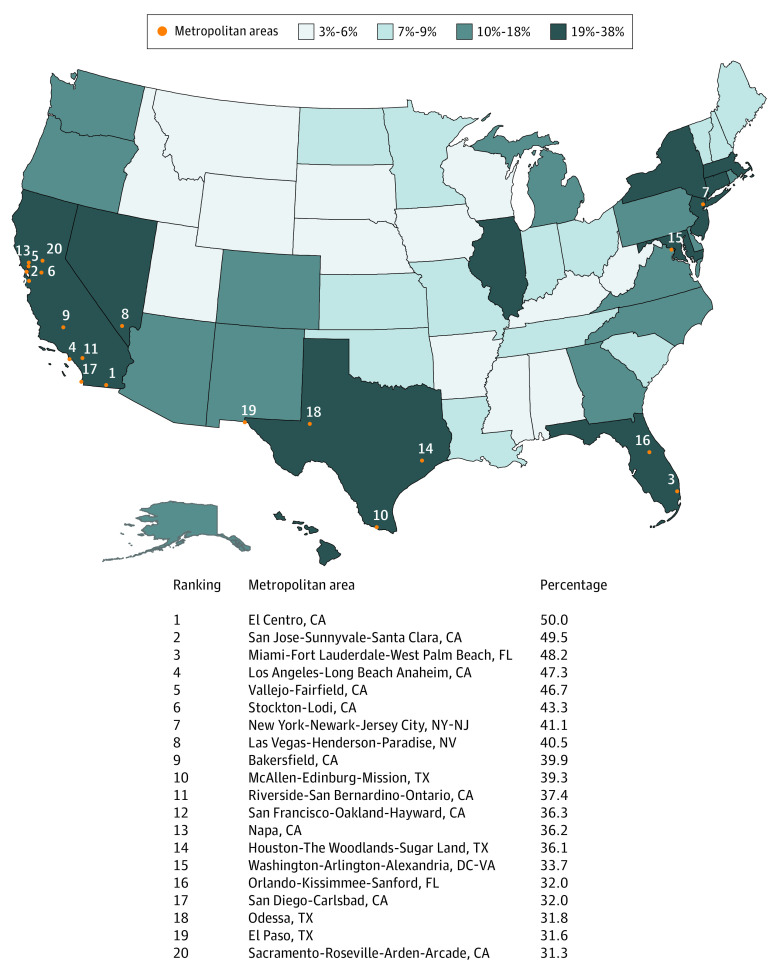
Percentage of Non–US-Born US Health Care Professionals by State, 2014-2018

### Non–US-Born Percentage of US Health Care Workforce

Rates of non–US-born HCPs differ by profession (Table 2). Approximately 1 in 3 physicians (30.0%; 95% CI, 29.6%-30.4%) and 1 in 6 of RNs (17.1%; 95% CI, 16.9%-17.3%) were non–US-born, while only 1 in 10 of APRNs were non–US-born (10.2%; 95% CI, 9.5-10.8). While the percentage of non–US-born HCPs has increased during the past decade (16.9% in 2010-2012 vs 17.6% in 2016-2018; *P* < .001), the percentage who have been in the US less than 10 years has decreased, from 3.0% between 2010 and 2012 to 2.6% between 2016 and 2018 (*P* < .001).

**Table 2.** Non–US-Born Percentage of US Health Care Workforce by Profession and Survey Period^a^

Characteristic	% (95% CI)		
	Overall	<10 y stay	≥10 y stay
**Profession**			
All HCPs (N = 657 455)	17.3 (17.2-17.4)	2.7 (2.7-2.8)	14.6 (14.5-14.7)
Physicians (N = 78 557)	30.0 (29.6-30.4)	4.9 (4.7-5.1)	25.1 (24.7-25.4)
APRNs (N = 13 509)	10.2 (9.5-10.8)	0.5 (0.4-0.6)	9.6 (9.0-10.3)
PAs (N = 8797)	13.9 (13.0-14.9)	2.0 (1.6-2.4)	11.9 (11.0-12.8)
RNs (N = 293 293)	17.1 (16.9-17.3)	2.8 (2.7-2.8)	14.3 (14.1-14.5)
LPNs/LVNs (N = 64 428)	15.1 (14.7-15.5)	2.4 (2.3-2.6)	12.6 (12.3-13.0)
Other HCPs (N = 251 857)	14.8 (14.6-15.0)	2.3 (2.2-2.3)	12.5 (12.3-12.7)
**Survey period**			
2010-2012	16.9 (16.7-17.1)	3.0 (2.9-3.1)	14.0 (13.8-14.2)
*F*_1 657 455_	[Reference]	[Reference]	[Reference]
*P* value	[Reference]	[Reference]	[Reference]
2013-2015	17.4 (17.2-17.6)	2.7 (2.6-2.8)	14.7 (14.5-14.9)
*F*_1 657 455_	8.72	16.75	27.30
*P* value	.003	<.001	<.001
2016-2018	17.6 (17.4-17.8)	2.6 (2.5-2.7)	15.0 (14.9-15.2)
*F*_1 657 455_	21.63	31.59	60.69
*P* value	<.001	<.001	<.001

Abbreviations: APRNs, advanced practice registered nurses; HCPs, health care professionals; LPNs, licensed practical nurses; LVNs, licensed vocational nurses; PAs, physician assistants; RNs, registered nurses.

^a^ Weighted using American Community Survey sampling weights, 2010-2018. Significance levels were adjusted for multiple comparisons using the Benjamini-Hochberg procedure; all *P* values were considered significant at <.05 the Benjamini-Hochberg critical value using a 5% false discovery rate and 6 contrasts.

### Associations Between Nativity Status and Employment Characteristics

Non–US-born HCPs with less than 10 and 10 or more years of stay worked 32.3 hours (95% CI, 19.2-45.4) and 71.6 hours (95% CI, 65.1-78.2) more annually than US-born HCPs, respectively (Table 3). Compared with US-born HCPs, non–US-born HCPs with less than 10 years of stay and 10 or more years of stay, respectively, were also 4.8% (95% CI, 4.2%-5.4%) and 2.0% (95% CI, 1.8%-2.3%) more likely to work at night; 20.3% (95% CI, 19.5%-21.1%) and 7.8% (95% CI, 7.3%-8.3%) more likely to reside in a MUA/P; and 4.7 (95% CI, 4.0%-5.4%) and 0.4% (95% CI, 0.03%-0.7%) more likely to work in the skilled nursing/home health settings than US-born HCPs.

**Table 3.** Adjusted Association Between Nativity Status and Employment Characteristics by Health Care Occupation in the 2010 to 2018 American Community Survey^a^

Group	Annual work hours, No.		*P* value	Works nights, %^b^		*P* value	Resides in a MUA/P, %^c^		*P* value	Works in skilled nursing/home health setting, %		*P* value
	Mean	Difference (95% CI)		Estimated total	Difference (95% CI)		Estimated total	Difference (95% CI)		Estimated total	Difference (95% CI)	
All HCPs (n = 657 455)												
US-born	1870.6	[Reference]	[Reference]	8.3	[Reference]	[Reference]	55.0	[Reference]	[Reference]	12.8	[Reference]	[Reference]
Non–US-born, <10 y	1902.9	32.3 (19.2 to 45.4)	<.001	13.1	4.8 (4.2 to 5.4)	<.001	75.3	20.3 (19.5 to 21.1)	<.001	17.5	4.7 (4.0 to 5.4)	<.001
Non–US-born, ≥10 y	1942.2	71.6 (65.1 to 78.2)	<.001	10.4	2.0 (1.8 to 2.3)	<.001	62.8	7.8 (7.3 to 8.3)	<.001	13.1	0.4 (0.03 to 0.7)	.02
Physicians (n = 78 557)												
US-born	2485.4	[Reference]	[Reference]	1.8	[Reference]	[Reference]	67.1	[Reference]	[Reference]	0.5	[Reference]	[Reference]
Non–US-born, <10 y	2560.1	74.7 (36.6 to 112.9)	<.001	2.1	0.3 (−0.3 to 0.8)	.33	66.2	−0.9 (−3.0 to 1.1)	.37	0.2	−0.3 (−0.5 to −0.1)	.003
Non–US-born, ≥10 y	2485.4	0.05 (−19.4 to 19.5)	>.99	1.8	0.0 (−0.3 to 0.3)	.89	57.4	−9.8 (−11.0 to −8.6)	<.001	0.5	0.00 (−0.1 to 0.14)	.99
APRNs (n = 13 509)												
US-born	1980.3	[Reference]	[Reference]	1.5	[Reference]	[Reference]	55.1	[Reference]	[Reference]	2.5	[Reference]	[Reference]
Non–US-born, <10 y	1921.5	−58.8 (−198.9 to 81.3)	.41	1.5	0.5 (−2.9 to 3.0)	.97	74.2	19.1 (8.6 to 29.7)	<.001	1.5	−0.9 (−3.9 to 2.0)	.51
Non–US-born, ≥10 y	1993.2	12.9 (−28.1 to 53.9)	.54	2.9	1.3 (0.2 to 2.4)	.02	60.4	5.3 (1.6 to 9.1)	.005	3.6	1.1 (−0.1 to 2.4)	.08
PAs (n = 8797)												
US-born	1995.1	[Reference]	[Reference]	2.3	[Reference]	[Reference]	60.6	[Reference]	[Reference]	0.8	[Reference]	[Reference]
Non–US-born, <10 y	1896.8	−98.4 (−221.5 to 24.8)	.12	4.3	2.1 (−1.1 to 5.3)	.21	82.4	21.8 (15.5 to 28.1)	<.001	1.0	0.3 (−1.2 to 1.8)	.74
Non–US-born, ≥10 y	1960.4	−34.7 (−91.4 to 22.0)	.23	2.3	0.0 (−1.1 to 1.1)	.10	67.1	6.4 (2.1 to 10.8)	.004	1.4	0.6 (−0.4 to 1.6)	.24
RNs (n = 239 293)												
US-born	1791.9	[Reference]	[Reference]	14.1	[Reference]	[Reference]	53.1	[Reference]	[Reference]	14.1	[Reference]	[Reference]
Non–US-born, <10 y	1806.39	14.4 (−3.7 to 32.6)	.12	25.3	11.2 (9.9 to 12.4)	<.001	78.4	25.2 (24.0 to 26.5)	<.001	20.4	6.2 (5.1 to 7.4)	<.001
Non–US-born, ≥10 y	1859.1	67.2 (57.9 to 67.5)	<.001	19.4	5.3 (4.7 to 5.9)	<.001	64.5	11.3 (10.5 to 12.2)	<.001	16.3	2.2 (1.6 to 2.9)	<.001
LPNs/LVNs (n = 64 428)												
US-born,	1754.8	[Reference]	[Reference]	10.5	[Reference]	[Reference]	43.9	[Reference]	[Reference]	46.1	[Reference]	[Reference]
Non–US-born, <10 y	1702.1	−52.7 (−96.2 to −9.2)	.02	12.5	1.9 (0.01 to 4.0)	.048	78.3	34.4 (31.9 to 37.0)	<.001	60.4	14.2 (11.3 to 17.2)	<.001
Non–US-born, ≥10 y	1795.0	40.2 (18.7 to 61.8)	<.001	13.2	2.7 (1.6 to 3.8)	<.001	60.0	16.2 (14.3 to 18.0)	<.001	51.1	4.9 (3.2 to 6.6)	<.001
Other HCPs (n = 251 857)												
US-born	1803.8	[Reference]	[Reference]	4.7	[Reference]	[Reference]	56.3	[Reference]	[Reference]	7.2	[Reference]	[Reference]
Non–US-born, <10 y	1801.9	−1.9 (−23.1 to 19.3)	.96	6.1	1.4 (0.7 to 2.2)	<.001	74.7	18.4 (17.0 to 19.7)	<.001	11.7	4.5 (3.7 to 5.5)	<.001
Non–US-born, ≥10 y	1851.1	47.3 (36.9 to 57.6)	<.001	5.7	1.0 (0.6 to 1.3)	<.001	63.8	7.5 (6.7 to 8.3)	<.001	7.4	0.3 (−0.2 to 0.7)	.22

Abbreviations: APRNs, advanced practice registered nurses; HCPs, health care professionals; LPNs, licensed practical nurses; LVNs, licensed vocational nurses; MUA/P, medically underserved areas/populations; PAs, physician assistants; RNs, registered nurses.

^a^ Statistics based on an inverse probability weighted sample. Inverse probability weighting was used to balance characteristics between the US- and the non–US-born. The characteristics associated with being US-born (vs non–US-born <10 years stay and non–US-born ≥10 years stay) included gender, age, highest educational degree, census region, residence in a metropolitan area, and year group (2010-2012, 2013-2015, and 2016-2018). Predicted margins for US-born vs non–US-born groups in US <10 years and ≥10 years were estimated using the margins command in STATA version 16; 95% CIs were estimated using the δ method. Significance levels were adjusted for multiple comparisons using the Benjamini-Hochberg procedure; all *P* values were considered significant at <.05 using a 5% false discovery rate and 8 contrasts.

^b^ An individual was classified as working at night if they reported arriving at work between 6:00 PM and 1:59 AM.

^c^ MUA/P county-level records were obtained from the Health Resources and Services Administration^19^ and matched to the American Community Survey data set based on county of residence using a crosswalk file from Integrated Public Use Microdata Series USA.^20^

Differences across professions were found. Among non–US-born physicians, only those with less than 10 years of stay worked more (74.7 hours, 95% CI, 36.6-112.9 hours), whereas those with 10 or more years of stay were 9.8% less likely to reside in MUA/P (95% CI, −11.0% to −8.6%) and those with less than 10 years of stay were 0.3% less likely to work in skilled nursing/home health settings (95% CI, −0.5% to −0.1%) than US-born physicians. Non–US-born PAs with less than 10 (21.8%; 95% CI, 15.5%-28.1%) years of stay and APRNs with less than 10 (19.1%, 95% CI, 8.6%-29.7%) and 10 or more years of stay (5.3%; 95% CI, 1.6%-9.1%) were more likely to reside in MUA/P than their US-born peers, and APRNs with 10 or more years of stay were also more likely to work at night (1.3%; 95% CI, 0.2%-2.4%). Among RNs and LPN/LVNs, inferences were the same as those obtained in the total sample except that only non–US-born RNs with 10 or more years of stay worked more per year (67.2 hours; 95% CI, 57.9-67.5 hours) than US-born RNs and non–US-born LPNs/LVNs with less than 10 years of stay worked 52.7 hours *less* (95% CI, −96.2 to −9.2 hours) per year than US-born LPNs/LVNs.

## Discussion

This analysis of national data offers fresh evidence that non–US-born HCPs (physicians, APRNs, PAs, RNs, LPNs/LVNs, and other HCPs) provide valuable skills and services in our health care workforce. During the study period, 17.3% of all working HCPs were non–US-born. These estimates are slightly higher than the analyses of ACS data by Patterson et al,^25^ which found that 15.7% of HCPs were non–US-born from 2011 to 2013. Non–US-born HCPs worked more hours per year, were more likely to work at night, reside in MUA/P, and work in the skilled nursing/home health setting than US-born HCPs. Also, since non–US-born HCPs also speak languages other than English, they possess language skills to improve the care of diverse patients. While 78.3% of US-born HCPs were women, only 68.3% of non–US-born HCPs were women, which may be explained by gender-based educational opportunities and skills-based migration policies that favor men.^26^ The percentage of non–US-born HCPs in major metropolitan areas in California, Texas, New York, and New Jersey was almost twice the percentage nationwide. Notably, these metropolitan areas were hard-hit during the peak of the COVID-19 pandemic in 2020.^8,27,28^

Studies have shown that immigrants to the US tend to be highly educated.^1,29^ Thirty-seven percent of non–US-born HCPs had at least a master’s degree compared with 27.2% of US-born HCPs. The Immigration and Naturalization Act of 1965^30^ has contributed to the presence of highly educated immigrants who are accepted through temporary visa programs for high-skilled workers. However, “brain-waste,”^31^ severe waste of human capital resulting from the unemployment or underemployment of highly skilled college-educated immigrants, is common. The nonrecognition or discounting of foreign academic credentials and the costly processes for obtaining US-based credentials limit the effective integration of non–US-born HCPs.^31^ Policies to eliminate brain waste among non–US-born HCPs may strengthen the health workforce.

While the proportion of non–US-born HCP increased over the study interval, there was a significant decline in the proportion of recently immigrated non–US-born HCPs (<10 years of stay). This trend mirrors national estimates, showing that legal migration to the US declined 7.3% from 2016 to 2018.^32^ Processing delays and policy changes, including “heightened screening and vetting”^33^ processes have been blamed for the decline in employment-based immigration.

Non–US-born HCPs were more likely to come from Southeast Asia (24.6%) than other world regions. Most HCPs from Southeast Asia were RNs and born in the Philippines. The sizable presence of Filipino RNs is no accident. In addition to the Exchange Visitor Program,^34^ the implementation of American nursing programs in the Philippines in 1898 resulted in an “Americanized” nursing curriculum taught in English.^35^ Thus, Filipino nurses are actively recruited to fill the shortage of US nurses.^36^ During the COVID-19 pandemic, about a third of nurses who have died from COVID-19 have been Filipino nurses, although they make up only 4% of the nursing workforce.^37^

The Hispanic population constituted about 18% of the US population and surpassed 60 million in 2019,^38^ yet they are underrepresented in US-born (5.9%) and non–US-born HCPs (15.5%). Hispanic people are less likely to receive preventive health care or guideline-based health care than non-Hispanic people because of several factors, including low rates of health insurance and poor access to affordable care.^39^ The COVID-19 pandemic has further amplified these longstanding health disparities among racial/ethnic minority groups, including Hispanic individuals.^15,16^ Given the benefits of culturally appropriate care, urgent policies are needed to increase Hispanic HCP representation.

Importantly, these results suggest that non–US-born HCPs play a vital role in improving health care access to reduce health disparities in several key ways. First, non–US-born HCPs, regardless of length of stay, worked more hours per year than US-born HCPs, and these results were driven primarily by physicians and RNs who worked more hours annually than their US-born peers. Non–US-born HCPs, especially those with less than 10 years of stay, were more likely than US-born HCPs to reside in MUA/P. Approximately 14 million Americans reside in MUA/P.^40^ Furthermore, as of December 31, 2020, there were 7290 primary medical health professional shortage areas, with almost 83 million Americans residing in these areas.^40^ It is estimated that 15 193 practitioners are needed in areas with shortages to fill primary care gaps, which are more prominent in states such as California, Texas, and Florida.^40^ We have shown that non–US-born PAs, APRNs, RNs, and LPNs/LVNs, regardless of length of stay, are more likely to reside in MUA/P than their US-born peers.

Although all non–US-born HCPs were more likely than US-born HCPs to work in the home health settings, these findings were driven mainly by non–US-born RNs and LPNs/LVNs with less than 10 years of stay. In 2016, over 8.3 million people received long-term care (LTC) services through nursing homes, home health agencies, adult day services, and assisted living facilities.^41^ Approximately 1.5 million nursing personnel, including RNs and LPNs/LVNs, work in LTC and support frail older adults and younger adults with disabilities to improve or maintain their physical function.^41^ However, the demand for LTC exceeds the current supply of caregivers. It is projected that the number of Americans needing LTC will double from 12 million in 2020 to 24 million by 2030.^42^ As the US population continues to age, demand for these services will grow considerably and will require adequate staffing of these facilities.

Non–US-born HCPs face unique challenges in the health care workforce. They may be more likely to face discrimination because of their race, gender, ethnicity, English proficiency, and accents. A prior study of non–US-trained nurses found that 40% reported experiencing discrimination in shift assignments, wages, or benefits. Those recruited from low-income countries were more likely to report these discriminatory experiences than other non–US-born nurses or US-born nurses.^43^ Similarly, international medical graduates have reported discriminatory experiences even when they possess the required education, training, skills, and ethics.^44^ Although bias against international medical graduates may stem from the misconception that they provide lower-quality care simply because of their national origin, they outperform US medical graduates on the Internal Medicine In-Training Examination^45^ and provide similar care compared with US medical graduates.^46^

Non–US-born HCPs were more likely to work at night than US-born HCPs. Among RNs, approximately a quarter of non–US-born RNs with less than 10 years of stay worked at night compared with 14.1% of US-born RNs. Immigrants are more 15.7% more likely to work the “graveyard shift”—from 6 pm until 8 am—and 25.2% more likely to work weekends than US-born persons.^47^ In the same study,^47^ non–US-born HCPs were 20.6% more likely to work unusual hours during the workweek than their peers after adjusting for sociodemographic factors. These nontraditional working hours may provide the added benefit of higher pay, family care flexibility, and childcare costs. Together, these findings highlight that non–US-born HCPs ensure that essential and night shifts are covered to avoid health care interruptions.

Non–US-born HCPs are often underutilized because of red tape in the credentialing process. However, during the peak of the COVID-19 pandemic in summer 2020, New York State relaxed the rules barring graduates of foreign medical schools from practicing medicine.^48^ This decision allowed these qualified physicians to help in the fight against COVID-19. Furthermore, the US State Department issued guidance in April 2020 calling for foreign medical professionals with approved visas or certificates of eligibility for exchange visitor programs to make appointments at their nearest embassy to expedite processing, “particularly those working to treat or mitigate the effects of COVID-19.”^49^ Provisions have been made to allow medical residents on J-1 visas to extend their US stay. These policies demonstrate the vital role of non–US-born HCPs in filling critical gaps in health care during a global pandemic. France has rewarded foreign-born HCPs on the frontlines of the COVID-19 pandemic by granting them citizenship.^50^ The US immigration system should prioritize attracting non–US-born HCPs^51^ and implement policies to acknowledge their commitment to the nation.

### Limitations and Strengths

This study had several limitations. First, analyses could not specifically examine those non–US-born HCPs who were trained outside the US, as that distinction is not made in ACS data. Instead, nativity status was used as the exposure. Second, employment characteristics were self-reported and may be subject to reporting error. Third, the 11 regions of birth include rich cultural diversity, which may have been lost when the data were aggregated. Fourth, PUMAs were used to categorize MUA/P in the ACS PUMS data set because census tract–level information was not available.

This study also had several strengths. First, a nationally representative data set was used that included reliable and timely sociodemographic information on HCPs that is generalizable to HCPs in the US. Second, the sample included diverse HCPs representing all world regions of birth.

## Conclusions

This cross-sectional study found that non–US-born HCPs constitute a growing segment of the health workforce. In major metropolitan areas, as many as 40% of HCPs are non–US-born. They work more hours annually, work more night shifts, tend to reside in MUA/P, and do more work in home health settings compared with US-born HCPs. Their invaluable contributions preceded the COVID-19 pandemic and will outlive the current crisis. Policy makers need to consider the characteristics and contributions of non–US-born HCPs in allocating scarce human resources to improve the health of Americans.
